# Glycemic Patterns Revealed by Continuous Glucose Monitoring in Patients with Type 2 Diabetes Undergoing Intermittent Hemodialysis: A Pilot Study

**DOI:** 10.3390/medsci14020324

**Published:** 2026-06-16

**Authors:** Miguel Angel Cuevas-Budhart, Joel Salvador Becerra-Barrera, Rogelio Iván Silva-Rueda, Daniela Vallejo-Avalos, Maricruz Ponce-Villavicencio, María Begoña Ilabaca Avendaño, Marcela Ávila-Díaz, Ramón Paniagua

**Affiliations:** 1 Unidad de Investigación Médica en Enfermedades Nefrológicas del Centro Médico Nacional Siglo XXI, Instituto Mexicano del Seguro Social, Mexico City 06720, Mexico; angel_budhart@hotmail.com (M.A.C.-B.); cramav@gmail.com (M.Á.-D.); 2 Nephrology Department, Unidad Médica de Alta Especialidad del Hospital de Especialidades Bernardo Sepúlveda Gutiérrez, Centro Médico Nacional Siglo XXI, Instituto Mexicano del Seguro Social, Mexico City 06720, Mexico; becerrabarrerajs@outlook.es (J.S.B.-B.); dr.silva.nefrologo@gmail.com (R.I.S.-R.); mponce3623@gmail.com (M.P.-V.); 3 Facultad de Estudios Superiores Iztacala, Universidad Nacional Autónoma de México, Tlalnepantla de Baz 54090, Mexico; danielavallejoavalos@gmail.com; 4 Nephrology Department, Hospital General de Zona No. 2A Venados, Instituto Mexicano del Seguro Social, Mexico City 15850, Mexico; jauregui0526@att.net.mx

**Keywords:** continuous glucose monitoring, diabetes mellitus type 2, glycemic variability, hemodialysis, time in range

## Abstract

**Introduction:** Glycemic control in patients with type 2 diabetes mellitus undergoing intermittent hemodialysis represents a clinical challenge. The pathophysiological alterations inherent to chronic kidney disease (CKD) and the dialysis procedure limit the usefulness of traditional metrics. In this context, continuous glucose monitoring (CGM) enables dynamic assessment of glycemic profiles and can reveal patterns of dysglycemia that go undetected in routine clinical practice. **Methods:** An observational, cross-sectional, and analytical pilot study involved 10 patients from the hemodialysis (HD) unit. CGM was carried out for 14 days. A paired analysis was performed to compare glycemic parameters on days with and without HD. Statistical evaluation was performed using the Shapiro–Wilk test and Student’s *t*-test; a *p*-value < 0.05 indicated statistical significance. **Results:** Time in range (TIR) showed considerable interindividual variability (24–100%), with hyperglycemia being the predominant factor. During HD sessions, glucose levels showed a marked intradialytic decline followed by incomplete post-dialysis recovery, a pattern that differed from non-dialysis days (paired *t*-test, *p* < 0.001; *n* = 10 paired observations). These findings should be interpreted as exploratory. Hypoglycemic episodes were infrequent, whereas persistent hyperglycemia prevailed. **Conclusions:** CGM reveals metabolic dysregulation frequently overlooked by traditional indicators such as glycated hemoglobin (HbA1c). These exploratory findings suggest that CGM may provide clinically relevant information in this population, although larger studies are needed before therapeutic recommendations can be established.

## 1. Introduction

Type 2 diabetes mellitus is the leading cause of end-stage kidney disease (ESKD) and remains highly prevalent among patients undergoing hemodialysis (HD) [1,2,3]. In this population, adequate glycemic control is considered a central component of clinical management. It has been consistently associated with a reduced risk of cardiovascular complications, fewer hospitalizations, and lower mortality [4].

Alterations in glucose metabolism in advanced chronic kidney disease (CKD) are driven by multiple pathophysiological mechanisms, including decreased renal insulin clearance, uremia-induced insulin resistance, and hormonal changes associated with HD [5,6,7]. During HD, growth hormone levels are reduced, while glucagon levels remain markedly elevated and the normal reciprocal relationship between glucagon, insulin, and glucose concentrations is impaired [8]. These factors contribute to high glycemic variability (GV), characterized by pronounced glucose declines during dialysis sessions and persistent postprandial hyperglycemia [3,9,10,11].

Traditional assessment of metabolic control using HbA1c presents significant limitations in these patients [2,10,12,13,14], factors such as anemia, the use of erythropoiesis-stimulating agents, and reduced erythrocyte lifespan, confound its clinical interpretation [6,12,15]. In this context, CGM allows for a more precise characterization of glycemic behavior by providing dynamic metrics, including time in range (TIR) and GV [11,16,17,18,19,20].

Although these metrics have been progressively incorporated into international guidelines [12,16], the evidence remains limited and heterogeneous for this population [6]. In particular, glucose kinetics throughout the dialysis session are not fully elucidated, nor is the extent to which insulin regimens reflect its fluctuations on days with and without HD [6,12,21,22,23,24]. This lack of standardization hinders the use of CGM to optimize therapeutic decision-making, leading to insulin regimens that often diverge from actual glycemic patterns [4].

In public healthcare settings such as the Mexican Social Security Institute (IMSS), where HD facilities are intensively used across multiple daily shifts (morning, midday, and late afternoon) and frequent schedule changes occur due to logistical demands, the burden of diabetes and ESKD is high [25]. Therefore, identifying these challenges is a clinical priority. The objective of this exploratory pilot study was to use CGM to evaluate glycemic patterns and their relationship with insulin regimens in patients with type 2 diabetes mellitus undergoing intermittent HD, comparing GV and TIR between dialysis and non-dialysis days, as well as in relation to meal schedules.

## 2. Materials and Methods

### 2.1. Study Design

An observational, cross-sectional pilot study was performed, incorporating prospective interstitial CGM over a 14-day period. The design enabled intraindividual analysis, with each participant serving as their own control. The study did not include therapeutic interventions or modifications to the patients’ usual treatment regimens. This study was conducted solely to explore the behavior of TIR and GV in this patient population.

### 2.2. Study Population

The study enrolled adult patients with insulin-dependent type 2 diabetic nephropathy who were undergoing HD as renal replacement therapy (RRT) and receiving insulin therapy at the Centro Médico Nacional Siglo XXI of the Mexican Social Security Institute (IMSS).

Participants were excluded if any of the following conditions were present: hospitalization within the previous 4 weeks for cardiovascular, metabolic, or infectious events; active infections; pregnancy; active malignancies; or positive serology for HIV, hepatitis B, or hepatitis C.

Participants with incomplete CGM records were also excluded from the analysis. Written informed consent was obtained from all participants prior to inclusion. The full 14-day monitoring period was completed by all enrolled participants, and usable data were obtained from each.

### 2.3. Continuous Glucose Monitoring

Each participant was fitted with Guardian™ 3 continuous glucose monitoring system (Medtronic Minimed, Northridge, CA, USA), which automatically recorded interstitial glucose every five minutes throughout the day. Monitoring was conducted for 14 consecutive days by sequentially applying two sensors, each with an approximate lifespan of seven days. Data were extracted and processed using the corresponding platform.

However, it is recognized that formal validation of the Guardian™ 3 sensor has not been specifically performed in patients undergoing hemodialysis. Factors inherent to chronic kidney disease, such as fluctuations in hydration status, changes in interstitial fluid composition, and peripheral microvascular alterations, may potentially affect sensor accuracy. Accordingly, the findings derived from this device should be interpreted with awareness of these limitations, and no claim is made regarding its formal validation in this particular population.

### 2.4. Study Selection

Sociodemographic and clinical data were collected, including age, sex, comorbidities, time on hemodialysis, as well as anthropometric and biochemical parameters, the latter were obtained at a single time point, specifically upon completion of the monitoring period. Insulin therapy regimens (insulin type, dose, and administration schedule) and meal timing were recorded. Meals were not standardized, and nutritional composition was not quantitatively assessed, as the study was intended to characterize glycemic patterns under routine clinical conditions rather than within a controlled dietary intervention. Food intake information was obtained based on patient self-report. The glucose concentration of the dialysate solution routinely used in the hemodialysis unit was 100 mg/dL.

Glycemic metrics included mean glucose and TIR, defined as the percentage of time with readings between 70 and 180 mg/dL. Hypoglycemic events were defined as interstitial glucose values below 70 mg/dL sustained for at least 15 consecutive minutes (three consecutive 5 min sensor readings), consistent with the Level 1 hypoglycemia threshold. Hyperglycemic events were defined as values above 180 mg/dL. For comparative analysis, records were grouped into HD days and non-HD days.

### 2.5. Statistical Analysis

Given the exploratory pilot nature of the study, the sample size (*n* = 10) was not based on formal power calculations but was considered appropriate for an initial exploratory assessment of glycemic patterns in this specific clinical population.

Quantitative variables were expressed as mean and standard deviation or median and interquartile range, depending on their distribution, which was assessed using the Shapiro–Wilk test. Qualitative variables were described as absolute frequencies and percentages.

For comparative analyses, CGM data were summarized into predefined matched intraindividual conditions (hemodialysis vs. non-hemodialysis days), rather than analyzing individual sensor readings as independent observations. For each participant, monitoring days were classified as HD days (*n* = 6 per patient) or non-HD days (*n* = 8 per patient), according to the individual dialysis schedule. In addition to the paired intraindividual comparison, the complete individual-level time-series data from each participant (5 min interstitial glucose readings across all available monitoring days) were used to calculate standardized CGM descriptive metrics: time in range (TIR, 70–180 mg/dL), time above range (TAR, >180 mg/dL), time below range (TBR, <70 mg/dL, and <54 mg/dL for level 2 hypoglycemia), mean glucose, standard deviation (SD), and coefficient of variation (CV%). These metrics were derived from the raw sensor output without interval ggregation and are reported as individual descriptive statistics. Cross-validation against pre-existing aggregated summary data confirmed agreement within ≤0.2 percentage points for all patients with available reference values. Each day was divided into three predefined matched 3 h intervals: pre-session (3 h before the start of the HD session or equivalent period on non-HD days), intrasession or equivalent (duration of the HD session, standardized to 3 h for non-HD matching), and post-session (3 h following the end of HD or equivalent). Within each interval, all available 5 min CGM readings were averaged to produce a single representative mean glucose value per patient per condition. The analytical unit for the paired comparison was therefore the participant-level mean glucose per interval per day type, yielding 10 paired observations per interval (*n* = 10). Intraindividual differences between HD and non-HD means were then compared using the paired Student’s *t*-test, as variables showed normal distribution.

### 2.6. Ethical Considerations

The study was approved by the National Research Ethics Committee (R-2023-785-072) and was conducted in accordance with current national regulations and the principles of the Declaration of Helsinki. It was considered a minimal-risk study because it involved only the subcutaneous placement of CGM sensors and the collection of routine clinical information. The authors declare that they have no conflicts of interest regarding the device or its manufacturer.

## 3. Results

Table 1 presents the sociodemographic characteristics of the study population (*n* = 10). The participants had a mean age of 57.6 ± 10.9 years, with a female predominance (70%). Half of the participants were married (50%), and their educational profile was less than or equal to high school. Regarding economic status, income was concentrated in the low middle (30%) and low (20%) ranges.

Table 2 summarizes the clinical and anthropometric characteristics. Vital signs remained stable, with no clinically significant abnormalities; mean blood pressure was 146.4 ± 20.4/80.6 ± 12.5 mmHg. The average BMI was 24.1 ± 3.4 kg/m^2^; 60% of patients were in the normal weight range, and 40% were overweight, with no cases of obesity.

Table 3 details the biochemical and hematological parameters. Laboratory findings revealed anemia (Hb 9.2 ± 1.2 g/dL; Hct 29.1 ± 4.0%) and hypoalbuminemia (3.0 ± 0.8 g/dL). A marked dispersion in fasting serum glucose levels was observed (152.7 ± 107.9 mg/dL), with a standard deviation approaching the mean value, suggesting substantial interindividual heterogeneity in glycemic control within the cohort. This finding reinforces the limitation of single-point glucose measurements in adequately reflecting metabolic stability in this population. Additionally, creatinine (7.0 ± 3.1 mg/dL) and urea (147.3 ± 57.4 mg/dL) levels are reported.

Table 4 reports individual patient characteristics, insulin regimens, and glycemic control. Details on the type of insulin, doses, and timing of administration are provided. Most patients received 3 h sessions, with one patient undergoing a 4 h schedule. TIR showed substantial inter-individual variability, ranging from 24% to 100%. Hyperglycemic episodes were recorded in the majority of patients, while hypoglycemic events occurred in a subset of the population with lower frequency relative to hyperglycemic events.

Table 5 presents the complete standardized CGM metric panel computed from the individual raw time-series data for each participant, including TAR, TBR (levels 1 and 2), CV%, mean glucose, and SD. At the group level, hyperglycemia was the predominant pattern (mean TAR 33.2 ± 27.2%), while level 1 hypoglycemia was present in only a minority of participants (mean TBR 2.0 ± 2.6%) and level 2 hypoglycemia was infrequent (mean TBR < 54 mg/dL: 0.2 ± 0.3%). Glycemic variability was high across the cohort (mean CV 31.9 ± 6.7%), with four participants exceeding the consensus threshold of 36% indicative of high variability.

**Figure 1:**
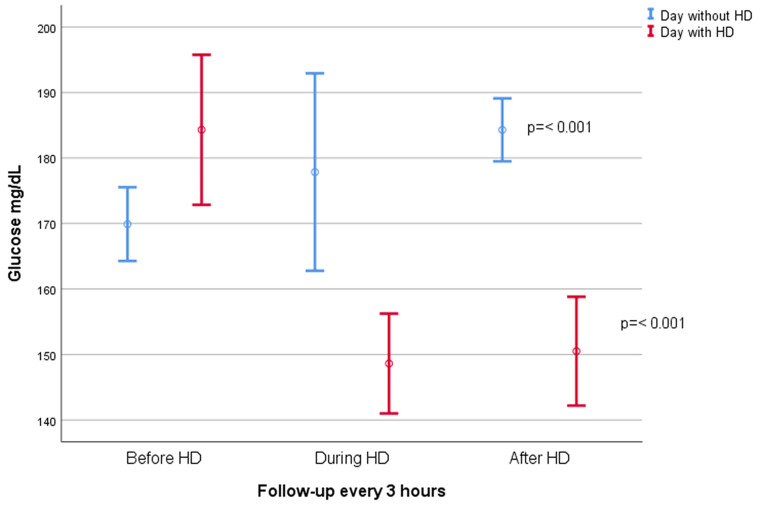
Comparison of glucose levels across matched time intervals on dialysis and non-dialysis days. Central markers represent mean glucose values, with error bars indicating 95% confidence intervals. Intraindividual comparisons were performed between dialysis days (red) and corresponding non-dialysis days (blue) using matched 3 h observational intervals before, during, and after hemodialysis sessions and equivalent time periods on non-dialysis days. Data were derived from 14 consecutive days of CGM for each participant (*n* = 10).

Figure 1 shows glucose variation every three hours before, during, and after HD, compared to days without treatment. On HD days, glucose levels decreased during the session and remained lower in the post-dialysis period. In contrast, on non-HD days, glucose levels followed a progressive increase across the same time points.

**Figure 2:**
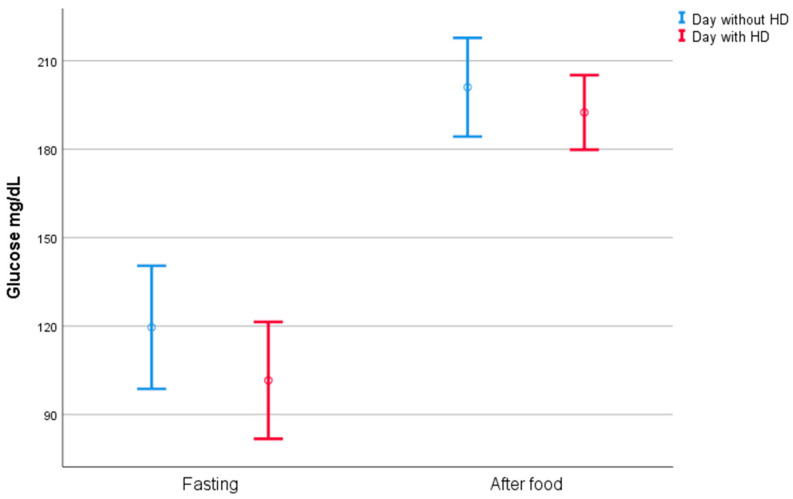
Comparison of glucose levels between fasting and postprandial states on days with and without hemodialysis. Data are plotted as mean glucose value with error bars denoting 95% confidence intervals. Intraindividual comparisons between fasting and postprandial states were performed using the paired Student’s *t*-test. Data were derived from 14 consecutive days of CGM for each participant (*n* = 10).

Figure 2 illustrates glucose variation according to dietary intake on days with and without hemodialysis. In both scenarios, glucose levels were higher after food intake compared to fasting. Distinct patterns between HD and non-HD days are evident, with lower glucose levels observed during and after dialysis.

**Figure 3:**
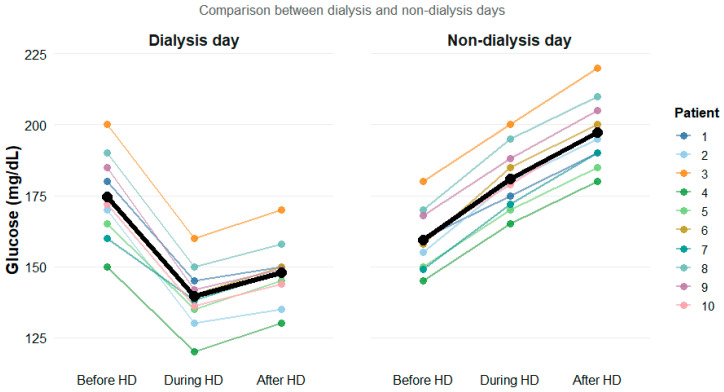
Individual CGM trajectories during dialysis and non-dialysis days. Colored lines represent individual participants, while the black line represents the overall mean glucose trajectory calculated from all participants at each corresponding time point *n* = 10). HD: hemodialysis.

Considerable interindividual variability in glucose behavior was observed across matched dialysis and non-dialysis conditions. Most participants exhibited a decline in glucose levels during hemodialysis, followed by incomplete post-dialysis recovery, whereas non-dialysis days showed a progressive increase in glucose levels over time (Figure 3).

## 4. Discussion

A central finding of the present study was the reduction in glucose levels on days with an HD session, followed by a slow and incomplete recovery towards normoglycemia. These findings should be interpreted as exploratory observations derived from a pilot cohort and should not be considered representative of the broader hemodialysis population. Although glucose levels decrease during the dialysis session, this effect is transient and does not offset the persistent postprandial hyperglycemia and incomplete recovery observed thereafter, resulting in an overall pattern characterized by high glycemic variability and a predominance of hyperglycemia. This pattern aligns with previous reports and has been attributed to partial insulin clearance during dialysis as well as to dynamic changes in insulin sensitivity [4,7,11]. However, the clinical relevance of these fluctuations remains a subject of debate, while some studies associate these declines with a higher risk of hypoglycemia, others report more heterogeneous patterns [4]. These patterns may be explained by underlying kidney insulin resistance, which disrupts normal glucose recovery mechanisms after dialysis [12].

The present study revealed marked discrepancies between CGM-derived glycemic behavior and conventional metabolic assessment in patients with type 2 diabetes mellitus undergoing intermittent hemodialysis. Despite insulin therapy, persistent hyperglycemia and reduced time in range predominated, suggesting that traditional markers such as HbA1c may inadequately reflect dynamic glucose fluctuations in this population. CGM enabled the identification of heterogeneous responses throughout the dialysis cycle, including intradialytic glucose decline and delayed post-dialysis recovery [4,5,26,27,28].

The persistence of postprandial hyperglycemia, both on days with and without HD, suggests that metabolic dysregulation is not merely a consequence of the acute effects of the dialysis procedure but is also consistent with, though not directly attributable to, the absence of dynamic insulin adjustment, as regimen data were not collected in a manner that permits causal inference. Previous studies have indicated that glycemic variability and prolonged exposure to hyperglycemia are associated with a poorer prognosis, independent of average glucose values [4,28,29]. This association is underpinned by insulin resistance-induced podocyte injury and renal lipotoxicity [8]. In this sense, the absence of hypoglycemia does not necessarily imply a favorable metabolic profile.

Furthermore, the glycemic dysregulation observed on HD days appears to be influenced by behavioral and therapeutic factors beyond intradialytic pathophysiological mechanisms. In routine clinical practice, carbohydrates of rapid absorption are frequently consumed by patients before, during, or immediately after dialysis. Consequently, significantly elevated glucose concentrations were exhibited during periods of food intake within this cohort. As previously described, the combination of post-dialysis caloric intake and conservative insulin strategies: frequently implemented to avoid hypoglycemia promotes prolonged hyperglycemic excursions and a subsequent reduction in TIR [6,11,23,27].

Additionally, the absence of documented insulin adjustments in the present study is consistent with previously described challenges in translating CGM-derived information into routine therapeutic decisions [6,19,20]. However, no direct assessment of clinical decision-making was performed in this study, and no causal inference can be drawn from these observations alone.

Although the potential value of this technology in patients with CKD has been recognized by international guidelines, specific recommendations for the population on HD remain limited and difficult to implement [18,21]. This absence of clear guidance is reflected in the heterogeneity observed in clinical practice and may account for the discrepancies reported across studies regarding the clinical benefits of this approach in this specific patient cohort.

### 4.1. Hypotheses for Future Research

The patterns observed in this pilot study generate hypotheses regarding the metabolic management of patients with type 2 diabetes mellitus on intermittent HD. The intradialytic glucose decline, coupled with delayed recovery and prolonged exposure to hyperglycemia, is consistent with a scenario in which fixed insulin regimens may not fully capture the glycemic variability induced by the dialysis session. These observations require prospective evaluation before any therapeutic inference can be drawn.

The coexistence of low TIR, persistent hyperglycemia, and a relatively low frequency of hypoglycemic episodes suggests the possibility of excessively conservative therapeutic strategies, aimed at preventing acute events at the expense of suboptimal metabolic control. In this context, CGM provided detailed characterization of glycemic patterns that are not consistently captured by traditional markers such as HbA1c.

### 4.2. Study Limitations

Although the pilot nature of the study and the modest sample size limit the generalizability of these observations, CGM enabled the identification of potentially relevant glycemic patterns. Further prospective studies with larger cohorts are warranted to confirm these hypothesis-generating findings. The re-analysis of individual raw sensor data enabled calculation of the full standardized CGM metric panel (TAR, TBR, CV%, and TIR) for all participants, reported in Table 5, and revealed a predominance of hyperglycemia (group mean TAR 33.2%) alongside substantial glycemic variability (group mean CV 31.9%). No definitive recommendations for clinical implementation or therapeutic adjustments can be made based on this pilot study alone.

Several limitations of this study should be acknowledged. The sample size was modest (*n* = 10), data were collected at a single center, and the observational design precludes causal inference. Dialysis-related variables (dialysate glucose concentration, ultrafiltration volume, meal timing, and insulin adjustments) were not prospectively standardized. The accuracy of the Guardian™ 3 CGM system may be affected by fluid shifts, altered tissue perfusion, and uremia-related physiological changes; caution is advised when interpreting individual glucose readings during sessions.

The initial paired analysis relied on interval-averaged data, which precluded calculation of standardized CGM metrics within that framework; re-analysis of the complete raw time-series resolved this, and the full metric panel is reported in Table 5. GMI and AUC were not calculated due to calibration and wear-time requirements not uniformly met. Future studies with larger cohorts, multicenter designs, and prospective metric collection are warranted to confirm these hypothesis-generating findings.

## 5. Conclusions

In patients with type 2 diabetes mellitus on intermittent hemodialysis, suboptimal metabolic control was revealed by continuous glucose monitoring (CGM), characterized by high glycemic variability and a predominance of hyperglycemia. These patterns were not reliably identified by traditional indicators such as HbA1c.

Hypoglycemic episodes, defined as CGM-recorded values below 70 mg/dL for at least 15 consecutive minutes, were identified in a subset of participants, though they occurred with substantially lower frequency than hyperglycemic episodes. Hyperglycemia remained the predominant glycemic pattern during the 14-day monitoring period. These findings should be interpreted cautiously and should not be considered representative of the broader hemodialysis population. The relatively low occurrence of hypoglycemia may reflect conservative insulin regimens rather than adequate metabolic control, particularly in the context of the marked intradialytic glucose decline and incomplete post-dialysis recovery observed (paired *t*-test, *p* < 0.001; *n* = 10 paired observations).

These findings should be considered hypothesis-generating. Although CGM appears to provide clinically relevant insights beyond acute event detection, larger prospective studies are warranted to confirm these observations and to better define the role of CGM in therapeutic decision-making for patients undergoing hemodialysis.

## Figures and Tables

**Table 1:** Sociodemographic variables.

Variable	HD *n* = 10
Age	57.6 ± 10.9
Gender	f (%)
Male	3 (30.0)
Female	7 (70.0)
Marital status	
Single	2 (20.0)
Marriage	5 (50.0)
Widowed	1 (10.0)
Common-law union	2 (20.0)
Schooling	
Elementary school	2 (20.0)
Middle school	3 (20.0)
High school	2 (10.0)
Technical/Vocational school	2 (20.0)
Undergraduate/Professional degree	1 (10.0)
Monthly Income	
Less than $2650	2 (20.0)
$2651–$5301	2 (20.0)
$5302–$7952	1 (10.0)
$7953–$10,603	3 (30.0)
$10,604–$13,254	1 (10.0)
More than $13,254	1 (10.0)

**HD**: Hemodialysis.

**Table 2:** Clinical and Anthropometric Characteristics.

Variable	Unit	Mean ± SD (*n* = 10)
Temperature	°C	36.3 ± 0.4
Heart Rate	bpm	76.2 ± 11.1
Blood Pressure	mmHg	
Systolic		146.4 ± 20.4
Diastolic		80.6 ± 12.5
Weight	kg	60.8 ± 11.2
Height	cm	158.3 ± 9.7
BMI	kg/m^2^	24.1 ± 3.4
Normal weight		6 (60.0)
Overweight		4 (40.0)

°C: Degrees Celsius, bpm: beats per minute, mmHg: millimeters of mercury, kg: Kilograms, cm: centimeters, BMI: Body Mass Index, kg/m^2^: kilograms per square meter.

**Table 3:** Biochemical and Hematological Parameters in Hemodialysis Patients (Mean ± Standard Deviation).

Variable	Unit	Mean ± SD
Serum glucose	mg/dL	152.7 ± 107.9
Creatinine	mg/dL	7.0 ± 3.1
Urea	mg/dL	147.3 ± 57.4
Cholesterol	mg/dL	142.0 ± 45.9
Albumin	g/dL	3.0 ± 0.8
Sodium (Na)	mEq/L	135.8 ± 3.9
Triglycerides (TG)	mg/dL	117.5 ± 46.5
Potassium (K)	mEq/L	4.9 ± 1.1
Chloride (Cl)	mEq/L	101.5 ± 5.7
Calcium (Ca)	mg/dL	8.2 ± 1.1
Phosphorus	mg/dL	4.8 ± 1.2
Hemoglobin (Hb)	g/dL	9.2 ± 1.2
Hematocrit (HTO)	%	29.1 ± 4.0
AST	U/L	35.3 ± 33.5
ALT	U/L	80.8 ± 121.7
Leukocytes	µL	7852.2 ± 3779.0
Lymphocytes	µL	1058.9 ± 633.4
Neutrophils	µL	5592.4 ± 3451.3
Platelets	mil/mm^3^	204.6 ± 63.1

AST: Aspartate Aminotransferase; ALT: Alanine Aminotransferase.

**Table 4:** Demographic Characteristics, Hemodialysis Schedule, and Glycemic Control of Included Patients.

Patient	Gender	Age	Hours HD	Days HD	Insulin Type	Dose	Unit	Timing of Administration	Schedule	Time in Range (%)	Hypoglycemic Episodes	Hyperglycemic Episodes
1	M	57	3	M-W-F	Glargina	30	IU	09:00	18:00–21:00	90	2	8
2	M	34	4	T-Th-S	Glargina	15	IU	21:15 ^a^	22:00–02:00	62	1	37
3	F	50	3	T-Th-S	Lispro	10	IU	23:00	10:00–13:00	100	-	-
4	M	61	3	M-W-F	Glargina	10	IU	21:00	14:00–17:00	24	-	76
5	F	59	3	M-W-F	NPH	10	IU	10:00 ^b^	18:30–21:30	88	7	5
6	F	60	3	M-W-F	Glargina	22	IU	09:00	14:30–17:30	41	-	59
7	F	62	3	T-Th-S	Glargina	8	IU	21:00	12:00–15:00	85	3	12
8	F	68	3	M-W-F	Glargina	12	IU	09:00	18:00–21:00	39	-	61
9	F	74	3	T-Th-S	Glargina	10	IU	21:00	18:00–21:00	46	1	53
10	F	51	3	T-Th-S	Glargina	20	IU	08:00 ^c^	06:00–09:00	71	7	22

HD: Hemodialysis; IU: International Units. ^a^: Patient on a dual-insulin regimen, with the second dose consisting of NPH insulin (10 IU) administered at 15:00; ^b^: Patient on a dual-insulin regimen, with the second dose consisting of NPH insulin (5 IU) administered at 23:00; ^c^: Patient received insulin during the hemodialysis session.

**Table 5:** Individual standardized CGM metrics derived from raw 5 min sensor data across all monitoring days (*n* = 10).

Patient	Monitoring Days	Sensor Readings (*n*)	Mean Glucose ± SD (mg/dL)	CV (%)	TIR (%) 70–180 mg/dL	TAR (%) > 180 mg/dL	TBR (%) < 70 mg/dL	TBR L2 (%) < 54 mg/dL
1	12	2598	132.9 ± 33.3	25.0	90.8	7.6	1.6	0.0
2	8	1506	163.2 ± 50.1	30.7	62.9	36.4	0.7	0.0
3	9	1354	115.2 ± 27.7	24.1	99.5	0.1	0.4	0.0
4	12	2221	247.9 ± 78.3	31.6	24.2	75.8	0.0	0.0
5	5	951	198.3 ± 50.5	25.5	41.1	58.9	0.0	0.0
6	14	3130	204.3 ± 62.8	30.7	40.3	59.7	0.0	0.0
7		536	116.8 ± 43.5	37.3	84.9	11.9	3.2	0.0
8	8	1766	119.4 ± 35.0	29.3	88.2	5.3	6.6	0.7
9	15	2536	140.1 ± 60.5	43.2	70.1	23.5	6.3	0.8
10	5	820	194.8 ± 80.6	41.4	46.2	52.9	0.9	0.0
Group X ± SD	10.2 ± 4.0	1942 ± 832	163.3 ± 45.9	31.9 ± 6.7	64.8 ± 25.9	33.2 ± 27.2	2.0 ± 2.6	0.2 ± 0.3

CGM: continuous glucose monitoring; CV: coefficient of variation; TIR: time in range; TAR: time above range; TBR: time below range; TBR L2: level 2 time below range; SD: standard deviation. Metrics were calculated from individual raw 5 min interstitial glucose readings without interval aggregation. Cross-validation against pre-existing aggregated reference values confirmed agreement within ≤0.2 percentage points for all patients with available reference data.

## Data Availability

The original contributions presented in this study are included in the article. Further inquiries can be directed to the corresponding author.

## Abbreviations

The following abbreviations are used in this manuscript:

CGM	Continuous Glucose Monitoring
HD	Hemodialysis
TIR	Time in Range
GV	Glycemic Variability
HbA1c	Glycated Hemoglobin
CKD	Chronic Kidney Disease
ESKD	End-Stage Kidney Disease
RRT	Renal Replacement Therapy
IMSS	Instituto Mexicano del Seguro Social
